# The Effect of Exercise and Learning Therapy on Cognitive Functions and Physical Activity of Older People with Dementia in Indonesia

**DOI:** 10.1155/2021/6647029

**Published:** 2021-08-04

**Authors:** Neti Juniarti, Ihda Al'Adawiyah MZ, Citra Windani Mambang Sari, Hartiah Haroen

**Affiliations:** ^1^Community Health Nursing Department, Faculty of Nursing, Universitas Padjadjaran, Bandung, Indonesia; ^2^H. Bakri Hospital, Kota Sungai Penuh, Jambi, Indonesia

## Abstract

**Background:**

This study aims to analyze the effect of exercise and learning therapy on the cognitive functions and daily physical activities of older people with dementia in Indonesia.

**Methods:**

This was an experimental study with a pretest-posttest design and a control group. Samples were selected using nonrandom sampling methods and were then randomly assigned to intervention and control groups. The study population was older people with mild-to-moderate dementia, and the sample number was 90 people. The intervention group received an Indonesian physical exercise program for older people and reading therapy through 12 sessions over four weeks. The intervention was led by a community health volunteer who has been trained and certified.

**Results:**

The mean score for cognitive function in the intervention group showed significant increase between pre- and postintervention, with *p* value < 0.001, and there was no significant difference in the control group before and after intervention, with a*p* value of 0.198. Further, the Mann–Whitney test showed that there were significant differences in the mean scores for cognitive function between the intervention and control groups with *p* value < 0.001 and a 95% confidence level.

**Conclusion:**

Based on the results, the Indonesian older people exercise program and reading aloud activity had a positive effect on the cognitive function of older people with dementia.

## 1. Introduction

The population with dementia worldwide in 2015 reached 46.8 million and is expected to increase to 75 million in 2030 and 135 million by 2050 [1]. The number of people with dementia is expected to increase from 960,000 in 2013 to 1.89 million in 2030 and to 3.98 million in 2050 [2]. Nearly 60% of people with dementia worldwide are found in poor and developing countries [1]. The prevalence of dementia in East Asia increased from 4.98% in 2009 to 6.99% in 2015 [1]. In Indonesia, there were 1.2 million people with dementia in 2016 [3].

Older people with dementia usually have symptoms such as impairment of daily memory, concentration, planning or regulating, language abilities, visuospatial abilities, and orientation [1]. As dementia progresses, older people might have difficulty in meeting their basic needs, thereby increasing their dependency ratio in performing daily activities [4]. Decreased cognitive function in older people is also influenced by lifestyle factors and brain stimuli [5], including lack of cognitive and physical activity, impaired social relationships, and unhealthy diet [6].

The literature review conducted for this study found that physical activity and cognitive therapy are the most frequently studied interventions for dementia and showed positive results for reducing risk of and treating dementia in older people [7–10]. The administration of more than one treatment is believed to provide optimal results in older people with dementia because compared with a single treatment, multiple treatments can improve several effects of dementia, such as cognitive function, performance of daily activities, depression, performance, and quality of life [11–14].

One type of aerobic exercise for older people is the Indonesian older people exercise program that involves simple movements and low-impact exercise with mild-to-moderate intensity [15]. This exercise program also poses low risk of injury for older people [16]. Physical exercise designed for older people could be a low-impact aerobic exercise with mild-to-moderate intensity that has simple movements and does not pose a risk of injury [17]. Research has found that physical exercise may be sufficient to overcome the increase of apolipoprotein E4, which is a predisposing factor in developing dementia [18]. In addition, research has found that physical exercise improves the cognitive abilities of older people, particularly in the part of the brain that is responsible for executive control, and that physical exercise increases the volume of the hippocampus, which plays a major role in memory [17].

Cognitive training programs are also used to treat dementia because they are designed to activate the mind, thereby improving cognitive function and quality of life for older people [19]. The types of activities for cognitive therapy are diverse because in principle, all activities that stimulate brain functions are classified as cognitive therapy. A study of older people with dementia concluded that audible reading (reading aloud activity) has the potential to stimulate the prefrontal area [20]. The results of this study proved that the cognitive function of the older people in the intervention group was better compared with the control group. In addition, storytelling activities, listening, and concluding the story were believed to have improved cognitive function, particularly in the areas of executive function and attention [13, 21].

There is a great deal of research on cognitive therapy, but most of this research compares cognitive therapy with other treatments such as physical activity, memory therapy, or occupational therapy [9, 12, 13, 22]. In general, the advantages of administering more than one type of intervention include attaining maximum effectiveness and efficient results, and it is appropriate for preventive and curative treatment; however, no study has examined cognitive therapy combined with other treatments in the same group [11]. By combining exercise and reading aloud activities, older people are able to maintain cognitive function and daily activities. Thus, the aim of this study was to investigate the effect of the Indonesian older people exercise program (low-impact aerobics) and reading aloud on the cognitive function and performance of daily activities among older people with dementia.

## 2. Materials and Methods

### 2.1. Research Design

This study was conducted using an experimental design from April 9 to May 12, 2019. The participants were divided into two groups: the intervention group and the control group. The pretest for both groups employed the Mini-Mental State Exam (MMSE) and the Physical Activity Scale for the Elderly (PASE). The intervention group was given treatment in the form of the Indonesian older people exercise program and a reading aloud activity that involved loud reading, listening, writing, and concluding a story. The control group was involved in an older people daily activities program conducted by a local community health center (known as “puskesmas” in Indonesian). A posttest was administered after four weeks.

### 2.2. Sample

The study population was community-dwelling older people with dementia in the Dago Community, West Java, Indonesia. The inclusion criteria were the following: (1) older people with decreased cognitive function or dementia attending the Dago Village Dago Community Health Center, whose dementia status had been diagnosed by a certified professional from the community health center; (2) MMSE score of 19–26 (indicating mild-to-moderate dementia) and a total PASE score of ≥15. The exclusion criteria were if the older people were unable to read and/or write; unable to perform daily activities independently; or were experiencing health problems that create risk when performing physical activity with moderate intensity.

The sample size was 45 participants in each group and was calculated based on the following data in a similar study by Vreugdenhil et al. [20].

(1) $$\begin{array}{c} n_{1}=n_{2}={\left(\frac{\left(Z_{\alpha }+Z_{\beta }\right)S_{d}}{d}\right)}^{2}, \end{array}$$

where *n* = number of samples; *n*_1_ = intervention group; *n*_2_ = control group; *S*_*d*_ = standard deviation obtained by finding the variance of the two populations; *d* = average difference between the two groups; *Z*_*α*_ = alpha standard deviation/level of significance (type I error rate) = 5%, *Z*_*α*_ = 1,960 (from table); and *Z*_*β*_ = standard beta deviation/*β* error rate (type II error rate) = 20%, *Z*_*β*_ = 0.842 (power = 80%). Given the probability of a dropout sample (10%), five people were added to each sample group in the study. Thus, the total number of participants in the study was 90 older people: 45 in the intervention group and 45 in the control group. The dropout criteria included the following: (1) participant missed at least two sessions of the intervention program (intervention group); (2) participants with health problems or became ill during the intervention process (intervention group); (3) participants who were unwilling to participate in the posttest (intervention and control group); and (4) participants who resigned from the study (intervention and control group). This study was approved by the Research Ethics Committee, Padjadjaran University, Bandung (ethics number: 258/UN6.KEP/EC/2019).

**Figure 1:**
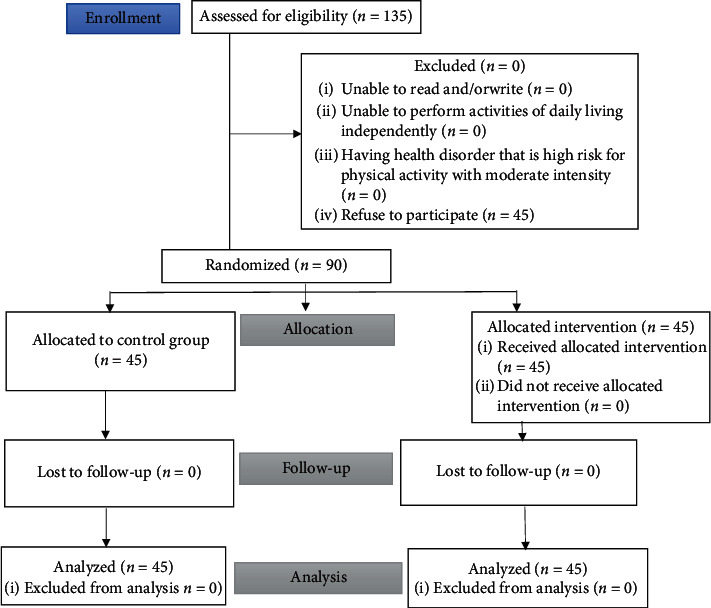
CONSORT flow diagram of participants.

The sampling technique used cluster sampling of ten clusters, chosen from a total of 17. Sample mapping and a CONSORT flow diagram of participants are presented in Table 1 and Figure 1, respectively.

### 2.3. Intervention Process

#### 2.3.1. Intervention Group Activity

Intervention activities were conducted for four weeks, from April 18 to May 10, 2019. The intervention was conducted in 12 sessions (three sessions per week for four weeks), and the duration of each session was 60 minutes. Intervention activities were led by 11 community health volunteers (Table 2) that were trained in administering the Indonesian older people exercise program and reading aloud activities. The criteria for the community health volunteers included availability to assist in the implementation of the study, junior high school as minimum educational level, attended training with 100% attendance, and fulfilled the competency indicator of training success (intervention group). There were no adverse events during interventions.

The procedure for implementing the intervention was achieved by dividing the participants into small groups of seven to eight older people, with each group then being led by one community health volunteer. The intervention began with a reading aloud activity for 30 minutes. In this intervention, the older people were asked to read a story that had been previously provided, conclude the story, provide responses to the content, and write their conclusions in the activity book. The steps in the reading aloud activity were as follows:

1. Reading activity: instructor provided an opportunity for group members to take turns reading the story aloud. The topic of the story was religious and health promotion activities for older people. The length of each story was a maximum of four pages, typed using Times New Roman size 12, with 1.5 spacing on an A4 size page with normal margins (approximately 2 cm).
2. Attention and listening activity: group members who did not get a turn to read aloud were asked to pay attention and listen attentively to the group members reading the story.
3. Writing activity: after completing the reading and listening activities, all group members were asked to write a short summary of the story.
4. Drawing conclusion activity: the instructor asked each member to draw their own conclusion to the story and explain the lesson learned from the story.

After the reading aloud activity, participants were given a five-minute break, and then the second intervention (i.e., the aerobic exercise) was administered. The Indonesian older people exercise program was led by the instructor for 30 minutes. The low-impact aerobic exercise program assigned to the participants consisted of warming up (5 minutes), core exercise comprising various aerobic movements (20 minutes), and cooling down (5 minutes). This exercise program involves low-impact repetitive movement without any rigorous movement such as jumping with a mild-to-moderate intensity [15, 23]. The movements consist of following steps:

Neck movement
  1. Stand straight and look straight ahead
  2. Lower the head slowly and then return to the original position
  3. Tilt the neck slowly to the left, center, and right
  4. Move the head slowly to the left, forward, and right
Shoulders and arms movements
  1. Turn the shoulders back and then forward.
  2. Straighten arms in front of the chest; move them inward and then sideways.
  3. Bent the arm parallel to the shoulder, move it in front of the chest, and pull it to the chestback. Do it alternately with left hand on top and right hand under.
Foot movements
  1. Walk in place with legs raised forward
  2. Cross leg steps to the right and left followed by a hand swing
  3. Lift the thigh and leg forward with an upward hand movement
  4. Move the right leg across in front, touch the tip of the right foot with left hand, and then do the opposite
  5. Move on tiptoe with toes
  6. Move the sole of the foot up with heel support and then lift the heel with toes support
  7. Bend the tip of the toes and then pull the tip of the toes up

All movements are done eight times count. These movements are repeated and easy to follow for older people. The exercise was accompanied by the music and movement instruction which has been designed for the aerobic exercise for Indonesian older people. Indonesian rhythmic music recording contained instructions for the movements. The exercise was monitored during the sessions for each participant. An example of Indonesian older people exercise program can be seen in the following link: https://www.youtube.com/watch?v=dKzGOv_ElLk.

During the study, no older people dropped out. Although two participants did not attend one session each for specific reasons, they were not excluded. In addition, the participants in the intervention group were enthusiastic about participating in the activity. The participants reported feeling happy to participate in the intervention and benefiting from the daily activities. After the intervention program was completed, the posttest was administered using the MMSE and PASE instruments in both the intervention group and the control group. These were administered by the researcher and a health professional from the community health center. The PASE was completed in an interview with the participants accompanied by family member for validation.

#### 2.3.2. Control Group Activity

The instructions for the participants in the control group included for them to conduct regular activity in the older people program that is offered by the local community health center, which consists of health checkups, health education, and once a month regular exercise. After four weeks, the posttest was administered using the MMSE and PASE instruments in the control group. At the end of the study (i.e., after the posttest was completed), the control group received the same treatment as the intervention group to ensure application of the justice principle for all participants.

### 2.4. Data Analysis

The skewness-kurtosis analysis technique was used to identify the normality of the data for the pretest and posttest. Data were analyzed using the Statistical Package for the Social Sciences (SPSS) version 22. The Mann–Whitney test was used to analyze the differences in cognitive function in both groups preintervention and postintervention because the data distribution was not normal. Moreover, the Wilcoxon signed-rank test was also used to analyze the differences in cognitive function preintervention and postintervention. The independent *t*-test was used to analyze the differences in physical activity for the intervention and control groups preintervention and postintervention because the data distribution was normal. In addition, the paired *t*-test was used to analyze the mean difference preintervention and postintervention. The degree of significance used was *p* − value < 0.05 with confident interval (CI) 95%.

## 3. Results

The participant characteristics recorded included age, gender, education level, occupation, marital status, home living arrangements, and homogeneity tests. There were 45 participants in each the intervention group and control group. Both groups were identical in almost all demographic characteristics except gender (Table 3). There were no significant differences between the two groups in relation to age (*p*=0.084); education level (*p*=0.092); occupation (*p*=0.286); marital status (*p*=0.187); and home living arrangements (*p*=0.448) preintervention, with distribution of gender being the only difference between the two groups (*p*=0.02). Thus, there were no significant differences in the demographic variables of the two groups apart from gender.

In the control group, there was no significant difference between the mean cognitive function scores pretest and posttest one month after the intervention (*p* > 0.05). In addition, there was no difference in the average score of physical activity between the pretest and posttest (*p* > 0.05) (Table 3). In contrast, in the intervention group, there was a significant difference between the average of these scores before test and one month after the intervention, with *p* value < 0.001(*p* > 0.05). The data also show that there were significant differences in the cognitive function scores before intervention and after intervention in the intervention group (Table 4).

In the intervention group, the increase in the average cognitive function score was 2.44, with a standard deviation of 2.408. However, in the control group, there was a decrease in the average cognitive function score of 0.18, with a standard deviation of 0.912. In addition, the mean difference between the groups was 2.62. The Mann–Whitney test *p* value was <0.01. Thus, it was concluded that there was an influence resulting from the aerobic exercise and reading intervention, indicated by a significant difference in the mean cognitive function scores between the intervention and control groups, with the mean difference being 2.62 at a 95% confidence level as presented in Table 5.

The increase in the average score of physical activity in the intervention group was 1.16, with a standard deviation of 1.127. However, in the control group, there was a decrease that averaged 0.11, with a standard deviation of 0.487. Meanwhile, the independent sample *t*-test revealed an influence resulting from the intervention, with a mean difference of 1.27 at the 95% confidence level (*p* < 0.001). Changes in the average score of cognitive function and physical activity in the two groups are presented in Table 6.

## 4. Discussion

This study found that combining low-impact aerobic exercise with reading aloud increased the older people 's cognitive function significantly compared with an exercise that was provided by Community Health Center. This finding is in line with the results of a meta-analysis of 18 randomized controlled trials that investigated the effects of physical activity on cognitive function in dementia patients, finding that this type of intervention positively influenced cognitive function in patients with dementia. These beneficial effects do not depend on the clinical diagnosis and frequency of interventions but, rather, were driven by the interventions, including aerobic exercise [20].

Further, the results demonstrate that low-impact aerobic exercise with mild-to-moderate intensity is safe and improves cognitive function in older people [24]. This means that this type of intervention plays a role in slowing the progression of cognitive decline in older people with dementia. In addition, low-impact aerobic exercise is conducted without any jumping movements, with one leg always remaining on the floor [24].

The increase in the average score of cognitive function in the intervention group resulting from the physical and mental interventions in the present study is in line with the findings and theories that have been demonstrated through previous research. For example, a study conducted in Korea that analyzed the effect of regular physical exercise in patients with dementia obtained a significant increase in the average cognitive function score (MMSE) (baseline: 14.53 ± 5.34, mid: 17.47 ± 6.90, and post: 19.07 ± 6.53) compared with the control group. This study tested cognitive function at baseline, six months into the intervention, and at 12 months (i.e., when the intervention was completed). The physical exercise interventions were conducted 2-3 times per week, with a duration of 30 to 60 minutes for 12 months [25].

Another study was conducted in Japan to determine the effect of reading aloud on brain function and activities of daily life in older people with a clinical diagnosis of dementia. The measurement of follow-up cognitive function scores (MMSE) in the intervention group was significantly higher (*p* < 0.05) than the control group [9]. The study concluded that early-stage rehabilitation interventions involving executive functions and aerobic training programs potentially improve memory function. Further, the study found that the number of interventions made a difference to cognitive function, while previous research had examined the effect of only one type of intervention in one participant group [9].

A study that reviewed several biomarkers of Alzheimer's disease, including brain-derived neurotrophic factor (BDNF) and tumor necrosis factor *α* (TNF-*α*), resulted in significantly lower BDNF levels in the early-onset Alzheimer's disease (EOAD) and the late-onset Alzheimer's disease (LOAD) groups compared with the control group (*p* < 0.05) [27]. In addition, TNF-*α* levels increased significantly in the EOAD and LOAD groups compared with the control group (*p* < 0.05) [26].

Cognitive values are associated with increased physical function [21]. In addition, the results of a systematic review in Australia concluded that physical activity positively affected the health and wellbeing of individuals living with dementia in nursing homes, particularly when multidomain approaches involved a combination of activities used in the intervention. This research demonstrated that engaging in physical exercise for at least 30 minutes twice a week produced significant improvements [27]. Although the PASE score was unable to predict a healthy physical size, the relationship between PASE and waist circumference is suitable for encouraging older people to be more active physically [28]. Other findings suggested that aerobic exercise alone does not improve executive function in dementia patients and that it has positive effects only on older people with no cognitive impairment [29].

In the present study, apart from being administered a low-impact aerobic exercise intervention designed for older people, the older people in the intervention group also participated in a reading aloud activity. The study implemented both these interventions to attempt to ensure a maximal increase in cognitive function in the older people with dementia. There was an increase of 2.62 points in the average cognitive function score in the intervention group after 12 sessions of the interventions over four weeks. The results obtained in this study showed that the use of more than one intervention can improve the cognitive function of older people with dementia. It is also important to note that application of more than one type of intervention has been found to result in different types of improvements in older people, for example, in cognitive function, daily activities, depression, performance, and quality of life [12–14].

Reading aloud activities are used as part of learning therapy in older people to improve cognitive capacity. Reading aloud involves a combination of several cognitive processes such as visual recognition of words, conversion from graphical to phonological representations of words, and pronunciation control [30]. A previous study related to the benefits of reading aloud activities in increasing cognitive abilities in older people with dementia concluded that the reading activity (stimulating reading) stimulates the prefrontal area [20].

The results of this study are in line with those of previous research that found older people with dementia experience sustained loss of cognitive function. The study found a global cognitive decline of 2.3 points on the MMSE over 12 months [31], meaning an average decline of 0.19 points every month [31].

The decrease in the average score of cognitive function in the control group found in the preset study was possibly caused by various factors, including lack of physical activity and inability to access regular cognitive stimulation. Based on the results of a meta-analysis of seven risk factors for dementia, the largest proportion of cases of Alzheimer's disease in the United States, Europe, and the United Kingdom was associated with a lack of physical activity [32]. Although for the participants in the present study, there were exercise programs for the community every Saturday in the residential area, the participants in the control group did not take part in the exercise regularly because there was no third-party monitoring as there was in the intervention group.

Previous findings have found a decrease in the average MMSE score in the control group from preintervention to postintervention [12], with the average MMSE score before and after the intervention at 14.20 and 13.80, respectively (*p*=0.65). This result is in line with a study of older people in Nagoya, Japan. Although respondents in the control group (older people with cognitive impairment) in this study were also administered an intervention to promote health, the MMSE showed a decrease in the average cognitive function score before intervention and after intervention of 26.7 and 25.8, respectively (*p*=0.032) [33].

This decline in cognitive function is unavoidable because the decline occurs because of physiological changes in the structure of the brain that usually occur with increasing age [34]. However, the older people in the control group did not participate in regular physical and cognitive activity therapy; thus, the decline in average cognitive function was natural but was not significant because the study period was only four weeks.

Research on a learning therapy intervention known as “Saido Learning”—a working memory training program that uses basic systematic problems in arithmetic and language, including reading aloud, and writing—found that the intervention group showed a statistically significant increase in cognitive function, as measured by the MMSE compared with the control group. In addition, a post hoc analysis showed that the Frontal Assessment Battery score at bedside of the intervention group increased [35].

The strength of the present study is that the fitness exercises and reading aloud intervention combined led by trained healthcare personnel not only improved cognitive function in older people with dementia but also increased their ability to participate in physical activity. This demonstrates that a short-term intervention can produce positive results in increasing the cognitive function scores and the ability to participate in physical activity in older people with dementia. However, long-term follow-up should be conducted to verify whether the positive changes in cognitive and physical abilities are long lasting. Although the MMSE has been widely used to assess cognitive function and has been proven effective in detecting cognitive impairment among older people, the limitation of this study is that the MMSE instrument is a subjective form of assessing cognitive function.

The main limitation of the study is that it is an experimental study conducted in a city in the region of West Java, which may have a different context from other regions in Indonesia, or from the wider global community. The older people being studied were in a single city and therefore the context may be limited to this particular region. However, this study can be used to inform other researchers who are interested in low-impact exercise for older people.

## 5. Conclusion

The results of this study demonstrate that implementing the reading aloud and Indonesian older people exercise program together led to a significant improvement in the cognitive function of the older people with dementia in this study. Further research is needed to review the long-term effects of the reading aloud and Indonesian older people exercise program interventions implemented in this study.

## Figures and Tables

**Table 1:** Number of research samples for each cluster.

Group	Cluster	Screened older people	Match the inclusion criteria	Number of samples
Intervention	Cluster 1	17	14	45
	Cluster 2	18	11	
	Cluster 3	10	5	
	Cluster 4	12	8	
	Cluster 5	12	7	

Control	Cluster 6	15	9	45
	Cluster 7	11	6	
	Cluster 8	16	12	
	Cluster 9	10	8	
	Cluster 10	14	10	

Total		135	90	90

**Table 2:** Characteristics of community health volunteers.

No.	Initial name	Age (years)	Education level	Experience
1	EM	44	Senior high school	10 years
2	NYR	45	Senior high school	8 years
3	EH	53	Bachelor	31 years
4	ICM	57	Senior high school	29 years
5	MK	36	Diploma	7 years
6	KK	37	Senior high school	1 year
7	EU	60	Senior high school	30 years
8	YR	48	Senior high school	10 years
9	SM	68	Junior high school	35 years
10	YM	45	Senior high school	7 years
11	R	38	Bachelor	3 years

**Table 3:** Frequency distribution of participants' characteristics and homogeneity results in the intervention group (*n* = 45) and control group (*n* = 45) in Dago Village Community Health Center working area.

Participants' characteristics	Intervention group(*n*=45)		Control group (*N*=45)		Total		*p* value
	*n*	%	*n*	%	*n*	%	
Age (years)							
60–70	32	71.1	28	62.2	60	66.7	0.084
>70	13	28.9	17	37.8	30	33.3	

Gender							
Male	10	22.2	15	33.3	25	27.8	0.021
Female	35	77.8	30	66.7	65	72.2	

Education							
Elementary school equivalent	30	66.7	36	80.0	66	73.3	0.092
Junior high school equivalent	6	13.3	3	6.7	9	10.0	
High school equivalent	8	17.8	5	11.1	13	14.4	
College	1	2.2	1	2.2	2	2.2	

Work							
Does not work	16	35.6	22	48.9	38	42.2	0.286
Laborer	7	15.6	5	11.1	12	13.3	
Housewife	10	22.2	7	15.6	17	18.9	
Entrepreneur/trader	9	20.0	6	13.3	15	16.7	
Public servant/pensioner	2	4.4	2	4.4	4	4.4	
Others	1	2.2	3	6.7	4	4.4	

Marital status							
Married	22	48.9	18	40.0	40	44.4	0.187
Widow/widower	23	51.1	27	60.0	50	55.6	

Home living arrangement							
Live alone	3	6.7	2	4.4	5	5.6	0.448
Live with a partner	7	15.6	7	15.6	14	15.6	
Live with children/grandchildren/family	35	77.8	36	80.0	71	78.9	

**Table 4:** Mean difference (mean) score of cognitive function and physical activity pretest and posttest in the control group in Dago Village Community Health Center working area.

Variable	Measurement	Mean	SD	*p* value
Cognitive function	Pretest	20.93	3.394	0.198^*∗*^
	Posttest	20.76	3.105	

Physical activity	Pretest	18.47	2.085	0.133^*∗*^
	Posttest	18.36	2.036	

^*∗*^Paired sample *t* − test.

**Table 5:** Mean difference (mean) score of cognitive function and physical activity pretest and posttest in the intervention group in Dago Village Community Health Center working area.

Variable	Measurement	Mean	SD	*p* value
Cognitive function	Pretest	22.49	2.753	<0.001^a^
	Posttest	24.96	3.309	

Physical activity	Pretest	18.56	1.984	<0.001^b^
	Posttest	19.71	1.950	

^a^Wilcoxon signed − rank test.^b^Paired sample *t* − test.

**Table 6:** Differences in the mean difference of cognitive function scores and physical activity pretest and posttest in the intervention group and control group in Dago Village Community Health Center working area.

Variable	Measurement	*N*	Mean	SD	Mean difference	*p* value
Average difference in cognitive function score	Intervention	45	2.44	2.408	2.62	<0.001^a^
	Control	45	–0.18	0.912		

Difference in average physical activity score	Intervention	45	1.16	1.127	1.27	<0.001^b^
	Control	45	–0.11	0.487		

^a^Mann–Whitney test. ^b^Independent sample *t* − test.

## Data Availability

Data are available upon request from the corresponding author.
